# Computational analysis of gene expression space associated with metastatic cancer

**DOI:** 10.1186/1471-2105-10-S11-S6

**Published:** 2009-10-08

**Authors:** Andrey Ptitsyn

**Affiliations:** 1 Center for Bioinformatics, Department of Microbiology, Immunology and Pathology, Colorado State University, Campus delivery 1682 Fort Collins, CO 80523, USA

## Abstract

**Background:**

Prostate carcinoma is among the most common types of cancer affecting hundreds of thousands people every year. Once the metastatic form of prostate carcinoma is documented, the majority of patients die from their tumors as opposed to other causes. The key to successful treatment is in the earliest possible diagnosis, as well as understanding the molecular mechanisms of metastatic progression. A number of recent studies have identified multiple biomarkers for metastatic progression. However, most of the studies consider only direct comparison between metastatic and non-metastatic classes of samples.

**Results:**

We propose an alternative concept of analysis that considers the entire multidimensional space of gene expression and identifies the partition of this space in which metastatic development is possible. To apply this concept in cancer gene expression studies we utilize a modification of high-dimension natural taxonomy algorithm FOREL. Our analysis of microarray data containing primary and metastatic cancer samples has revealed not only differentially expressed genes, but also relations between different groups of primary and metastatic cancer. Metastatic samples tend to occupy a distinct partition of gene expression space. Further pathway analysis suggests that this partition is delineated by a specific pattern of gene expression in cytoskeleton remodeling, cell adhesion and apoptosis/cell survival pathways. We compare our findings with both report of original analysis and recent studies in molecular mechanism of metastasis.

**Conclusion:**

Our analysis indicates a single molecular mechanism of metastasis. The new approach does not contradict previously reported findings, but reveals important details unattainable with traditional methodology.

## Background

The utility of microarrays in cancer research has been recognized as this technology became available. Early studies [1] exploring the benefits of simultaneous estimation of activity for thousands of genes have been followed by hundreds of publications reporting important discoveries in cancer biology. In the past decade, design of both experiment and analysis pipelines have settled into a "classic" template with certain variations reflecting specific goals of the study. Samples are collected from contrast groups, such as normal and cancerous tissue or primary and metastatic tumors and comparison is conducted between such groups. The entire data set is reduced to a smaller, more manageable number of genes (features), informative in comparing pre-defined classes. Statistically significant differential genes, after adjustment for possible false discovery rate (FDR), are selected for further analysis. Additionally, the whole set or a subset of selected genes are subjected to hierarchical cluster analysis to highlight the difference in expression pattern between classes of samples. The goal of cluster analysis in this case is to identify the subset of genes with the highest potential to serve as predictive biomarkers. Some papers follow an alternative approach and start the analysis with unsupervised clustering procedures [2-5]. The results show "molecular classification" of cancer into subtypes not necessarily following traditional histopathology classification. Studies show a difference in survival rate for the patients that belong to different subtypes. However, the nature of these subtypes has proved to be hard to interpret and even harder to bring into clinical practice. At least partially, the utility of molecular sub-typing of cancer samples is hampered by the intrinsic limitations of the analysis.

Classification is typically based on the set of pre-selected "informative" features. In turn, the "informative" property of each feature/gene is estimated by variability in the general population or between pre-defined classes of samples. This pre-selection introduces bias into the overall picture of gene expression. Rarely expressed and marginally differential genes tend to be filtered out before their relation to the biology of cancer could be established on the grounds of signal intensity and variation alone, without any consideration of correlated gene expression and gene interaction. As a result, the final interpretation of results is based on a biased subset of relatively highly expressed genes depleted of early switches and enriched with non-specific downstream effectors.

In earlier publications we proposed an unsupervised classification algorithm that does not require dimensionality reduction. The algorithm was tested on cancer gene expression data [6] and was essential for understanding of patterns of gene expression associated with progressive insulin resistance in skeletal muscle [7]. On the other hand, we have also proposed a new approach to the selection of biomarkers based on systems biology, allowing inclusion of marker genes less differential on their own, but closely interlinked in the context of biological pathways. A similar approach has also revealed important biomarkers for diagnosis of persistent bovine diarrhea [8]. However, these studies relied on pre-defined sample classification and didn't attempt any cluster analysis. This paper proposes to join high-dimension unsupervised clustering with a systems biology approach in attempt to elucidate the molecular mechanisms of metastasis.

Metastasis is the deadliest development in cancer, responsible for most cancer-related deaths. Metastatic cells acquire mobility and spread through the blood and lymphatic system to form colonies in other organs. There are multiple theories about the origin of metastasis [9-13], but our own previous studies suggest that primary and metastatic tumors share the same basic metabolism. Differences between primary and metastatic tumor samples compared across multiple tissues of origin are associated with cytoskeleton remodeling, antigen representation, extracellular matrix and some other pathways[14]. In this study, the data is limited to just one set of experiments comparing primary and metastatic samples of prostate cancer. The original publication of this data [15] presents the state of the art in both functional genomics and bioinformatics of cancer. However, it still leaves open the question we seek to solve in this paper.

## Results and discussion

**Figure 1:**
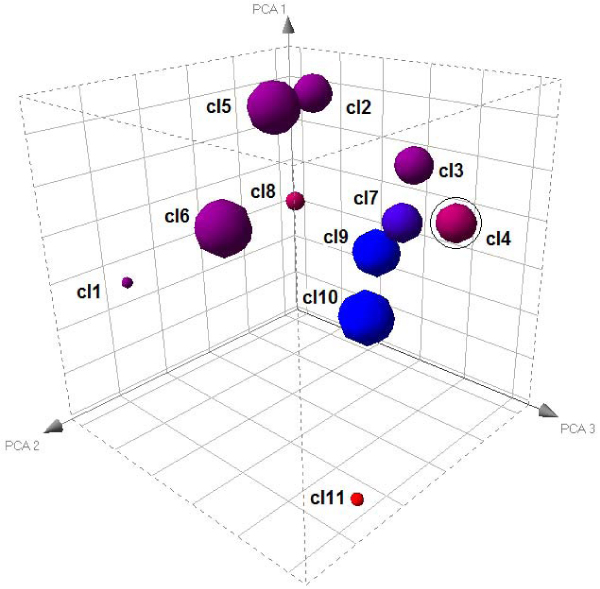
**Relative position of metastatic and non-metastatic cluster of samples**. Clusters 9 and 10 contain only metastatic samples (marked blue), Cluster 7 contains one metastatic and two non-metastatic samples. Clusters 1 and 11 are singletons.

The first inspection of the cluster analysis output produced expected results. Among nine clusters and two singleton listed in the FOREL output, two clusters were composed entirely of metastatic tumor samples and one contained two primary and one metastatic samples. This observation is generally consistent with the hierarchical clustering on the unreduced set of genes reported in the original publication of LaTulippe et al[15]. The patterns of gene expression in metastatic tumor samples are more similar within the class than they are to most non-metastatic samples. Further analysis reveals a few important details unattainable with the original analysis. The estimated relative position of clusters in gene expression space is depicted on Figure 1. The bulk of gene expression space defined by samples is occupied by variable size clusters of non-metastatic samples. Three clusters containing metastatic samples were situated close to each other and tended to occupy a definite area of elongated shape, which starts with the cluster containing mixed metastatic and non-metastatic samples.

The FOREL algorithm tends to break large uneven continuums into clusters the same way the human eye tends to separate constellations on the night sky (even though they in fact belong to the same Milky Way galaxy). This property is created by consecutive extraction of clusters from the overall population of samples rather than simultaneous partitioning (for example in k-means family of algorithms). On each step, FOREL identifies the best cluster, but clusters extracted on different steps are not necessarily separable from each other and can even overlap in space. This property of the algorithm creates the opportunity to probe the entire space of features (genes) for areas of higher or lower density, concentration or depletion of samples of certain class (metastatic, for example) and then reconstruct relative positions for such areas. Interpretation of cluster juxtaposition allows us to make a few important observations. The congregation of clusters made of non-metastatic samples occupies a large space and the clusters are not clearly separable from each other or forming any geometrical pattern. The observed "cloud" is consistent with a single class with relatively high variability. Three clusters containing metastatic samples are also likely to represent the same continuous class, but opposite to non-metastatic they occupy a compact area, show less variation and demonstrate an elongated pattern. In other words, metastatic tumors are more constrained in the ratios at which different genes can be co-expressed, possibly more tightly regulated and originating from a limited to a single origin and a single path of development. It remains for future studies to determine whether this elongated trend reflects the progression of metastatic transformation and can be used to estimate the age of the metastasis from the event that trigger the transformation. For resolution, these questions need more information than is contained in this data, but the missing data could be acquired in future experiments.

**Figure 2:**
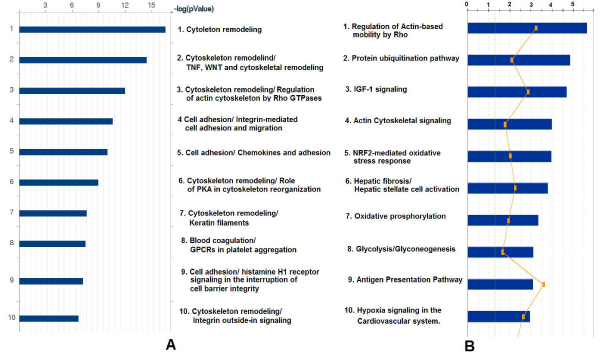
**Biological pathways separating metastatic and non-metastatic expression space as defined by GeneGo Metacore (A) and Ingenuity Pathway Analysis (B)**. Only 10 most significant pathways are shown (image is edited/stretched to fit the same dimension). The complete diagrams are available in supplementary materials (Additional file 1).

While the space of gene expression on Figure 1 is reduced to abstraction, direct comparison between selected areas (marked by clusters, cluster centroids and other landmarks) can be performed in the original unreduced space. Genes differentially expressed between such areas can be further studied using biological pathway and gene interaction tools. Interpretation of the pathway analysis results provides important clues about biology behind the patterns. The overview of biological pathways overrepresented among genes differentially expressed between primary and metastatic clusters is given in Figure 2. The complete chart of significant pathways can be found in supplemental materials (Additional file 1). This analysis shows both commonality and difference compared to meta-analysis of primary vs. metastatic tumors of different origin[14]. The oxidative phosphorylation pathway that dominates the lists of significant pathways in meta-analysis as well as colon and breast cancer sets is not near the top of the chart, but both oxidative phosphorylation and glycolysis/glyconeogenesis are reported among statistically significant pathways. On the other hand, a number of pathways related to cytoskeletal remodeling, tissue morphology, cell adhesion and cell motivity are highly significant in all studies.

**Figure 3:**
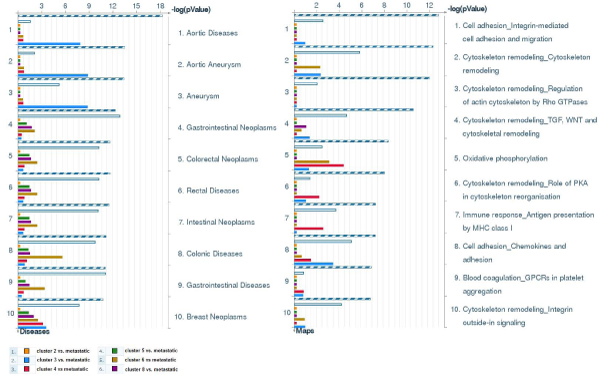
**Comparative analysis of biological pathways differential between metastatic and different areas of non-metastatic expression space**. Shared identical pathways correspond to the striped bars, similar pathways correspond to the empty white bar. Pathways unique to particular clusters are color-coded. For example, oxidative phosphorylation pathway contributes more to the difference between metastatic and Cluster 4 than any other cluster.

Using FOREL cluster centroids as anchor points, we can also compare metastatic samples to primary tumors, not as a whole homogeneous class, but as a conglomerate of subclasses. This comparison probes different areas of expression space flagged out by non-metastatic samples. Figure 3 shows comparison analysis between metastatic and different subspaces of non-metastatic area interpreted in terms of biological pathways. The bulk of biological pathways show exactly or approximately the same difference shared by all areas of non-metastatic space. However, there are genes unique to separate parts of the space and the number of such differences is consistent with the distance between clusters. Cluster 4 is the nearest to metastatic space, but it has differences in oxidative phosphorylation, cytoskeleton remodeling and immune response pathways in addition to all other differences shared among non-metastatic samples. These pathways are among most prominent general differences reported in meta-analysis of primary vs. metastatic tumors [14]. Proximity of Cluster 4 to the metastatic space and the pattern of gene expression characteristic to this space allow the hypothesis that there is an area in non-metastatic cancer expression space from which metastatic cells are recruited after metabolic transformation. Such transformation shifts the energy balance further towards glycolytic pathways in energy production (Wartburg effect [16]). Clusters observed in proximity to the metastatic expression space are separated from metastatic transformation by minimal changes in metabolism that can probably be induced by hypoxia or other conditions in growing primary tumors.

**Figure 4:**
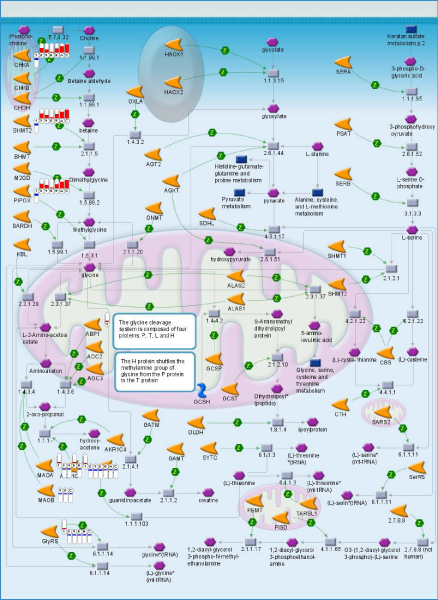
**Map of Glycine metabolism**. Gene expression pattern is marked by flags showing direction (blue-lowered, red – increased expression) and relative scale of change. Flag 1 corresponds to metastatic expression space, flags 2, 3, 4, 5, 6, A, B and C correspond to clusters 2,3,4,5,6 and 8.

Observations are not limited to the canonic pathways defined by the software developers like Ingenuity or Genego. In some cases, pre-defined pathways obstruct the analysis by including too many gene interactions related through shared genes or metabolites, but not necessarily acting as a whole in every biological process. It has been recently reported that sarcosine (N-methyl-glycine) can be a good predictor of metastatic prostate cancer [17]. Sarcosine level can be relatively easily estimated in a blood test. This biomarker has been identified in a large-scale proteomics study. However, glycine metabolism does not appear in the list of differential pathways in our study. The only reason for that the canonic map includes multiple chains of reactions and gene interactions involving glycine. A quick look at the map (Figure 4) shows that only the chain of enzymatic reactions leading from phosphocholin to glycin is involved. However, the entire chain is consistently down-regulated. Blockage of methylglycine demethylation can explain accumulation of sarcosine and it increased levels easily detectable in the bloodstream. The data we used for this analysis was published seven years ago and already contained all the clues pointing towards the role of glycine metabolism in metastatic development. With additional computational analysis and systems approach, this important discovery could have been made much earlier. Figure 3 also shows the difference in expression levels if metastatic samples are compared to different clusters of primary tumors and, thus, different areas of non-metastatic expression space. Notably, Cluster 4 has the least difference in expression levels in comparison to metastatic tumors. In Figure 1, Cluster 4 is also the nearest to the group of metastatic samples in expression space. This observation means that sarcosine accumulation in blood may be indicative of an aggressive tumor, but not all primary tumors are equally distinguishable from metastatic tumors by this test. On the other hand, topological (and thus metabolic) proximity of some primary tumors to the metastatic expression space may indicate higher potential for metastatic progression in the future.

## Methods

### Data

The original data set includes 3 non-cancerous, 23 primary tumor and 9 metastatic tumor samples collected at MSKCC between 1993 and 1999. Samples were snap-frozen in liquid nitrogen and stored at -80°C. Each sample was histologically examined. Cells of interest were manually selected from the frozen block, trimming away non-neoplastic tissues. RNA was extracted by homogenization using RNAeasy kif (Qiagen, Valencia, CA) and evaluated for integrity by denaturing agarose gel. Complementary DNA was synthesized from total RNA using t7-promoter-tagged oligo(dT) primer. Gene expression was estimated by hybridization to Affymetrix U95 microarrays. MAS 4.0 (average difference) algorithm was used to quantify expression values.

### Clustering algorithm

As a starting point for the algorithm development we took the heuristic concept proposed by Zagoruiko et al. [18,19], which included the original idea of a limited radius hypersphere, moving stepwise to the mass center of captured objects. This idea represents a departure from widely used k-means and other hypersphere-based algorithms. This algorithm has been also extensively discussed in a reference book for applied statistics in economics [20]. Since their first introduction algorithms of the FOREL family have been widely applied in taxonomic analysis of biomedical data, pattern recognition in geology and image analysis [21-24]. The original algorithm underwent a significant development to accommodate for extreme dimensionality of gene expression data. The results of this development and case study in classification of molecular subtypes of lung cancer has been recently published [6]. Our algorithm is based on dynamic amalgamation of objects (for example, expression profiles) in vicinity of an artificially introduced object (FORmal ELement). The vicinity is defined by equal distance from a point in all directions by selected inter-object distance metric (such as Euclidean, correlation, binary, etc.). Although theoretically the vicinity could be defined as any geometrical shape around the given point, only hyperspheric vicinity has been implemented and used in this study. FOREL clustering is based on the perception of the data set *O *as:

where *O*(*i*) is a cluster of n_*i *_elements. Clusters are extracted from the general population in order of their statistical fitness (see Cluster validation). This perception is fundamentally different from the popular *k-means *algorithm, which shares certain similarity with FOREL, but in k-means concept the whole data set is a sum of distinct classes rather then a union. FOREL clusters can partially or completely overlap in space or even share the same centroid, but can be separated as long as they differ in other statistical characteristics, for example density. In a nutshell, a white and a yolk of an egg would be separate classes by FOREL, while inseparable by *k-means*. Other hypersphere-based algorithms such as k-means imply Gaussian distribution of objects (phenotypes) in clusters [25]. FOREL is more flexible and does not require such assumptions. FOREL effectively combines the best features of k-means and hierarchical clustering approaches for the price of increased computation complexity. However, the performance of our C++ implementation is acceptable; up to a few hundreds of microarrays can be clustered on a PC within one hour. The algorithm starts with positioning of a hypersphere with a radius *R*_0 _and a center C_0 _in a certain coordinate, which can be one of the objects or a centroid of pre-defined cluster or any other point of interest in the expression space. Position of the "formal element" element is calculated as a center of mass of all objects, for which the distance *ρ*_*i*_(*C*_*i*_) ≤ *R*_*i*_. After the mass center of all captured objects is calculated, its center is moved to the new position. If new objects are found within the radius from the new position, they are added to the provisional cluster and the mass center is recalculated. This process is repeated until the no more objects can be added on the current step of the algorithm and the hypersphere stops.

### Cluster validation

Our version of FOREL consists of alternating steps of cluster isolation and cluster validation. Each completed walks of a hypersphere with *R*_*n *_and a center C_*m *_produces a provisional cluster *O(R*_*n*_*C*_*m*_), which is temporarily stored. We perform an exhaustive coverage of the data trying each element of the original data set as potential starting point. For each starting point we perform series of clustering steps with different hypersphere radius, ranging between minimum *R = min(D(C*_*i*_, *C*_*j*_))+*μ *and maximum *R = min(D(C*_*i*_, *C*_*j*_))-  Here *D(C*_*i*_, *C*_*j*_) is a distance (for example, Euclidean) between any two objects in the data set and *μ *is a margin, introduced to reduce computation time. The step of *R *increment is also a parameter. The resulting provisional clusters are fuzzy subsets, each captured by a hypersphere with specific radius as it moves gradually from the starting to the resting point. The validity of the provisional cluster can be verified by a statistical utility measure based on density, variance, sum of inter-cluster distances, etc (see [26] for review). If the cluster meets the selection criteria, it is removed from the original data set and the process reiterated until no more statistically valid (according to the chosen metric) unclassified objects are left or the best provisional cluster does not satisfy the minimal fitness requirement. Current implementation accommodates a few different metrics for computational cluster validation, but only two metrics have been applied in this study, density-based:

 if *n*_*i *_≥ 2 and *F *= 0 othersise;

and connectivity-based:

 for a cluster of *n *elements

This metric is a reasonable compromise between precision and performance, which has proven to be effective in analysis of microarray data [6].

Application of FOREL family algorithms has a number of potential pitfalls which have to be considered. Clusters produced by FOREL may overlap in space partially or completely, thus should not be assumed separable by ANOVA. In fact further analysis of relations between FOREL clusters may reveal continuous trend, like in was in case of gene expression patterns in skeletal muscle of diabetic and non-diabetic patients [7].

### Software implementation

The implementation developed by A. Ptitsyn [6] employs a complete test of every object as a possible cluster seed or hypersphere starting point. By default the current version of the program implements an iterative solution: all possible radii are tested with a certain step within a limited range. The step (or precision) is derived from the analysis of variation of distances within the whole data set. The range is defined by the minimal and maximal distance found within the whole dataset. These are extreme values, with a radius less than a minimal distance, the algorithms can produce only singletons, and on the other hand with radius equal to the maximal distance, all objects are guaranteed to fall into one large cluster. The best radius is one that produces a provisional cluster with the best quality. "Best" is an ambiguous term which can be defined differently in different versions of FOREL. Current implementation has a parameter which allows to choose between quality estimated through density or connectivity of cluster elements (see "Cluster validation"). Cutting percentile margins from the possible radius range can reduce the computation demands of the program. By default 20% of the range is cut from both minimal and maximal radius values. The "brute force" approach to computational cluster validation implemented in current version (see "Cluster validation") provides more reliable results compared to re-sampling, but results in considerably longer execution times. Typical running time for FOREL clustering does not exceed a few minutes on an average Pentium 4 PC. Depending on the validation metric applied and the parameter settings complete clustering of a large data set (up to a few hundred microarrays) data can take up to a few hours. The demand for computational power is significantly mitigated by effective C++ implementation and generally affordable, considering the time required to collect such data.

Current implementation of FOREL clustering algorithm runs on the standard PC under MS Windows (Win32 console application) or Linux. FOREL execution time is practically unaffected by the dimensionality of the data, but can be sensitive to the number of objects (samples) in the set.

### Cluster visualization

For cluster visualization dimensionality is reduced from over 12,000 down to 3 in two consecutive steps. First, the results of FOREL cluster analysis are used to identify centroids of non-singleton clusters. These points are used as anchors to reduce the dimensionality down to the number of identified clusters. Second, principal component analysis is applied to reduced the "centroid space" down to the space of three first principal components. Spotfire Decisionsite (TIBCO Software, Palo Alto, CA) is used for graphical depiction of clusters. The resulting picture reflects juxtaposition of clusters based on geometrical distances between cluster centroids and cluster radius. Individual position of samples inside each cluster is lost in dimensionality reduction, but guaranteed to be inside the space delimited by cluster radius.

### Selection of differentially expressed genes

A set of differentially expressed genes was selected using University of Pittsburgh Gene Expression Data Analysis suite (GEDA)[27]. The software is available for downloading from http://bioinformatics.upmc.edu/GE2/GEDA.html. For selection, we applied the standard J5 metric with threshold 4 and optional 4 iteration of Jackknife procedure to reduce the number of false-positive differential genes. Both J5 metric and threshold parameter are standard pre-set values recommended by the developers. We did not attempt to estimate the confidence level of individual genes and used J5 not as a statistical test, but as a selection procedure providing a shortlist of genes deviating from the expected average value and enriched with differential genes. This metric has obvious limitation and could not be recommended for a direct substitute of *t*-test. However, it is also free from assumptions of independence and normal distribution of intensities (or gene abundance estimates) required for *t*-test. Used for preliminary selection followed by computational validation and pathway analysis J5 produces unbiased and sensible results. The details of tandem application of preliminary J5 inference with selective pathway analysis are discussed in Ptitsyn et al [14].

### Functional annotation and pathway analysis

Analysis of biological pathways was performed using MetaCore software (GeneGo Inc.), Ingenuity Pathways Analysis (IPA, Ingenuity Systems Inc.). Significance of a particular pathway represented in a given list of genes is estimated by Fisher's exact test with adjustments to current database contents. The GeneGo and IPA databases are accessed online and the contents (including Canonic Maps, Molecular Functions, Gene Interactions etc.) are frequently updated by the corps of curators reading research publications and extracting information related to all forms of interaction among genes and chemical compounds. Consequently, the results of pathway analyses performed at different times may differ in details. The maps of canonic signaling and metabolic pathways are fuzzy, but tested for significance independently. As a result, the list of significant pathways often enriched with redundant pathways overlapping by majority of components. In comparison between such lists of pathways Metacore distinguishes "similar" and "identical" pathways as shown in Figure 3. The interpretation of the meaning of statistically significant pathways relies on the knowledge of the biological function underlying the pathway maps and cross-comparison between two independent pathway databases (IPA and Metacore).

## Competing interests

The author declares that she has no competing interests.

## Supplementary Material

Additional file 1 This file contains all supplemental materials referenced in the text in achieved (zip) format. Click here for file
