# Supplementary material for: Computational analysis of gene expression space associated with metastatic cancer
Source: BMC Bioinformatics. 2009 Oct 8;10(Suppl 11):S6. doi: 10.1186/1471-2105-10-S11-S6 (PMC3226195; doi:10.1186/1471-2105-10-S11-S6)
Supplement: Additional file 1 — This file contains all supplemental materials referenced in the text in achieved (zip) format. [file 1471-2105-10-S11-S6-S1.zip › MetaCore_legend.pdf]

Network Objects

Enzymes	
Generic enzyme	
**KINASE**	**PHOSPHATASE**
Generic kinase	Generic phosphatase
Generic protein kinase	Generic protein phosphatase
Generic lipid kinase	Generic lipid phosphatase
**PHOSPHOLIPASE**	
Generic phospholipase	
**PROTEASE**	**GTPase**
Generic protease	G-alpha
Generic metalloprotease	RAS - superfamily

Generic classes
Receptor ligand
Transcription factor
Protein
Cell membrane glycoprotein
Anchoring phospholipid
Molecule
Inorganic ion

G protein adaptor/regulators
G beta/gamma
Generic (RGS, GDI, GAP, GAF, GRF, ARF)
Heterotrimeric G-protein

Channels/Transporters
Generic channel
Ligand-gated ion channel
Voltage-gated ion channel
Transporter

Receptors
Generic
GPCR
Receptors with kinase activity
Nuclear receptor

Adaptors/regulators
Generic binding protein
Adaptor

Expression data

Overexpressed gene(s)
Genes with higher conditional expression level compared to the experimental "control"

Underexpressed gene(s)
Genes with lower conditional expression level compare to the experimental "control"

Mixed-expressed gene(s)
Genes with conditional expression level statistically different from the experimental "control", with the "sign" of expression varying in different experiments

Groups of objects

Group of related object(s)
Group of objects with common properties

Protein complex
Group of proteins physically connected in a complex

Group1 **User created group**
Group of collapsed objects chosen by user

Other marks

Red circle
The links terminated due to a restriction of the number of steps in network expansion.
Network may be expanded from such nodes

Blue circle
The links terminated due to network truncation.
Network may be expanded from such nodes

Object highlighting

Nodes and root nodes

Found object
Object selected on the search pane

Manually selected node(s)
Object(s) selected by ctrl + click on it or by click + drag rectangle around it

Highlight by mouse over

Highlight upstream objects
When the mouse is over an object (node on a network), the closest interacting nodes are highlighted in CYAN if the direction of interaction is **from** the initial object

Highlight downstream objects
When the mouse is over an object (node on a network), the closest interacting nodes are highlighted in yellow if the direction of interaction is **towards** the initial object

Root nodes

Root node(s) for network expansion (building)
Object(s) from a user-specified uploaded list or from experiments

Initial object(s)
Object(s) chosen to build the pathways **from**

Intermediate object(s)
Object(s) situated along the pathway

Terminal object(s)
Object(s) the pathways terminate

Possible combinations of three above marks (except the first one)

Connectors

Connectors

Incoming interaction
When the mouse is over an object, yellow link indicates direction to the object

Outgoing interaction
Cyan link indicates direction **from** the object

Bidirectional interaction
Blue link indicates **BI-DIRECTIONAL** interaction

Non-directional link
Blue link also indicates an interaction for which the direction is not specified

Traced link
The link is always highlighted in blue if both linked objects are selected in "Trace" mode

Custom marked links (user's choice)

Interactions between objects

Functional interactions

- **Positive effect**
- **Negative effect**
- **Unspecified effect**

Mechanisms

Direct interactions

A — **Allosteric regulation**
Compound binds to the allosteric site of enzyme in a non-covalent manner and alters it's activity

B — **Binding**
Compound binds the enzyme or receptor

C — **Cleavage**
Cleavage of a protein at a specific site yielding distinctive peptide fragments. Proteolytic cleavage can be carried out by both enzymes and compounds

CM — **Covalent modifications**
Protein activity regulation by covalent binding of a small chemical group to the aminoacids of an active site.

+P — **Phosphorylation**
Protein activity is altered via addition of a phosphate group

–P — **Dephosphorylation**
Protein activity is altered via removal of a phosphate group

T — **Transformation**

Tn — **Translocation**

Z — **Catalysis**

Tr — **Transcription regulation**

S — **Substrate**
Enzyme may attenuate the compound's regulatory effect by decreasing its level

PD — **Product**

Indirect interactions

IE — **Influence on expression**
Compounds change the expression level of target genes indirectly, for instance by binding to upstream receptors

? — **Unspecified interactions**
Mechanism is unknown

Logical relations

CR — **Class relation**
Object belongs to a generic group of related objects

CS — **Complex subunit**
Active protein is a subunit of a protein complex

(888)592-3124
www.genego.com
GeneGo
