# Supplementary figures and images for: Computational analysis of gene expression space associated with metastatic cancer
Source: BMC Bioinformatics. 2009 Oct 8;10(Suppl 11):S6. doi: 10.1186/1471-2105-10-S11-S6 (PMC3226195; doi:10.1186/1471-2105-10-S11-S6)

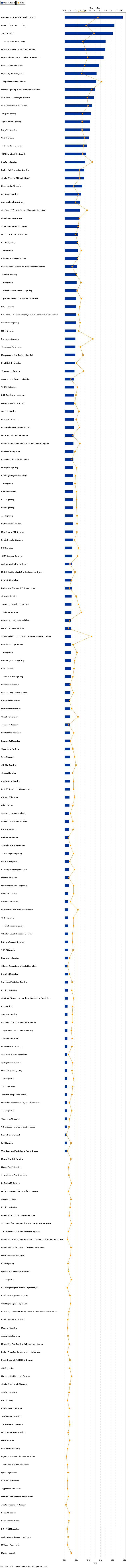

Supplement: Additional file 1 — This file contains all supplemental materials referenced in the text in achieved (zip) format. [file 1471-2105-10-S11-S6-S1.zip › distributionIPA.png]

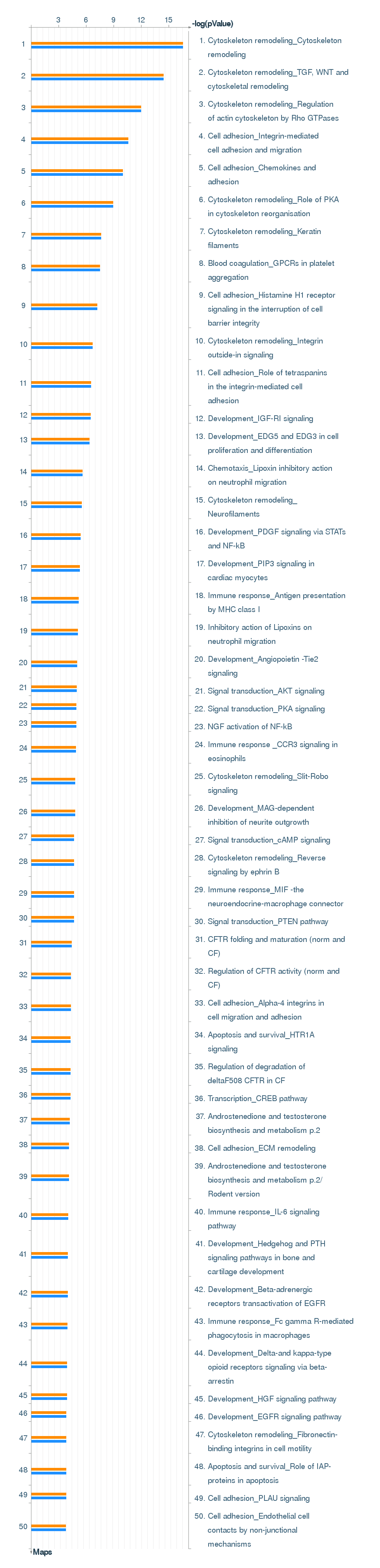

Supplement: Additional file 1 — This file contains all supplemental materials referenced in the text in achieved (zip) format. [file 1471-2105-10-S11-S6-S1.zip › distributionMetacore.png]
